# Precise tuning of the glyoxylate cycle in *Escherichia coli* for efficient tyrosine production from acetate

**DOI:** 10.1186/s12934-019-1106-0

**Published:** 2019-03-19

**Authors:** Minji Jo, Myung Hyun Noh, Hyun Gyu Lim, Chae Won Kang, Dae-Kyun Im, Min-Kyu Oh, Gyoo Yeol Jung

**Affiliations:** 1 School of Interdisciplinary Bioscience and Bioengineering, Pohang University of Science and Technology, 77 Cheongam-Ro, Nam-Gu, Pohang, Gyeongbuk 37673 South Korea; 2 Department of Chemical Engineering, Pohang University of Science and Technology, 77 Cheongam-Ro, Nam-Gu, Pohang, Gyeongbuk 37673 South Korea; 3 Department of Chemical and Biological Engineering, Korea University, 145 Anam-ro, Seongbuk-gu, Seoul, 02841 South Korea

**Keywords:** Acetate, Tyrosine, Glyoxylate cycle, Gluconeogenesis, Metabolic engineering, Synthetic biology

## Abstract

**Background:**

Acetate is one of promising feedstocks owing to its cheap price and great abundance. Considering that tyrosine production is gradually shifting to microbial production method, its production from acetate can be attempted to further improve the economic feasibility of its production.

**Results:**

Here, we engineered a previously reported strain, SCK1, for efficient production of tyrosine from acetate. Initially, the acetate uptake and gluconeogenic pathway were amplified to maximize the flux toward tyrosine. As flux distribution between glyoxylate and TCA cycles is critical for efficient precursor supplementation, the activity of the glyoxylate cycle was precisely controlled by expression of isocitrate lyase gene under different-strength promoters. Consequently, the engineered strain with optimal flux distribution produced 0.70 g/L tyrosine with 20% of the theoretical maximum yield which are 1.6-fold and 1.9-fold increased values of the parental strain.

**Conclusions:**

Tyrosine production from acetate requires precise tuning of the glyoxylate cycle and we obtained substantial improvements in production titer and yield by synthetic promoters and 5′ untranslated regions (UTRs). This is the first demonstration of tyrosine production from acetate. Our strategies would be widely applicable to the production of various chemicals from acetate in future.

**Electronic supplementary material:**

The online version of this article (10.1186/s12934-019-1106-0) contains supplementary material, which is available to authorized users.

## Background

Because of ethical and economic issues on the use of starch crop-based feedstock [1], alternative and abundant feedstock for microbial biochemical production are being extensively investigated [2–4]. In this regard, acetate is a promising feedstock as it is cheap and abundant. For example, acetate is inevitably generated as a byproduct during the pretreatment of lignocellulosic biomass [5]. In addition, acetate is a primary intermediate in anaerobic digestion of organic wastes [6, 7]. Furthermore, it is well known that acetate is produced as a major product of C1 gas (CO, CO_2_, and methane) fermentation [8–10]. While its utilization has been limited due to its toxicity to microorganisms [11], several recent studies successfully demonstrated the conversion of acetate into value-added chemicals [3, 12, 13], suggesting its great potentials as a feedstock.

Tyrosine is one of the amino acids and can be utilized as a precursor for the synthesis of flavonoids and alkaloids in food, pharmaceutical, and cosmetic industries [14, 15]. As the demand for tyrosine is increasing, methods for its efficient production are being investigated [16, 17]. Traditionally, tyrosine is extracted from raw materials of plant or animal origin [17]. However, the extraction method showed low yield [15, 17, 18] and unstable productivity due to compositional fluctuation of the raw materials [19]. Hence, microbial tyrosine production from biomass-derived sugar has been attempted as an alternative (Additional file 1: Table S1) [17, 20, 21]. In the early stage, less-rational approaches, including utilization of analogs [22–24] or construction of auxotrophs [25], were used to improve tyrosine production. Recent advances in microbial engineering have enabled the development of various rational approaches, including overexpression of pathway genes [26], de-regulation of feedback regulation [27, 28], and precursor balancing to maximize production [17]. These efforts have successfully demonstrated efficient tyrosine production by the engineered microorganisms.

**Fig. 1:**
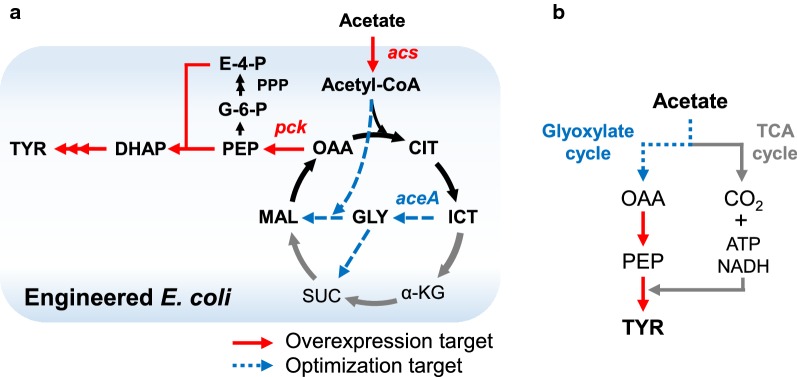
Overall strategy used in this study. **a** To produce tyrosine from acetate, the SCK1 strain, with amplification of the pathway from PEP to tyrosine, was used [17]. To further amplify the linear pathway from acetate to PEP, *acs* (encoding acetyl-CoA synthetase) and *pck* (encoding phosphoenolpyruvate synthase) were overexpressed. Thereafter, the glyoxylate cycle pathway was precisely controlled by varying the expression of *aceA* (encoding isocitrate lyase). **b** A schematic showing the pathway optimization strategy. Acetyl-CoA produced from acetate can be metabolized via two different pathways: oxidation into CO_2_ and generation of ATP and NADH via TCA cycle, or assimilation as metabolites and cell biomass via the glyoxylate cycle. Replenishment of precursors (PEP from OAA) and energy consumption for tyrosine production should be considered for efficient tyrosine production. This can be implemented by precise control of the glyoxylate cycle. *OAA* oxaloacetate, *CIT* citrate, *ICT* isocitrate, *α-KG* α-ketoglutarate, *SUC* succinate, *MAL* malate, *GLY* glyoxylate, *PEP* phosphoenolpyruvate, *G-6-P* glucose-6-phosphate, *E-4-P* erythrose-4-phosphate, *DHAP* dihydroxyacetone phosphate, *TYR* tyrosine

For efficient biochemical production, a linear pathway should be amplified to increase the carbon flux [29]. In addition, the overall pathway should be optimized to avoid shortage of precursor supplementation [17, 30, 31]. The generation of key precursors, namely, phosphoenolpyruvate (PEP) and erythrose-4-phosphate (E-4-P), is essential for efficient tyrosine production [20]. Although genes for PEP synthesis including *acs* (encoding acetyl-CoA synthetase), *maeB* (encoding NADP-dependent malic enzyme), *pck* (encoding PEP carboxykinase) [32] are up-regulated in acetate assimilation, there is still room for further enhancing metabolic flux by overexpression [12, 13]. Moreover, the activity of glyoxylate cycle can be crucially considered for efficient precursor supplementation (Fig. 1a, b). Acetate should be used for the replenishment of precursors via the glyoxylate cycle for tyrosine production [12, 32, 33]. Meanwhile, acetate has to be metabolized via the TCA cycle for ATP and NADH generation that are also used for tyrosine synthesis. Optimization of glyoxylate cycle activity is crucial as it determines the flux distribution between glyoxylate and TCA cycles [12, 34]. Consequently, glyoxylate cycle activity should be precisely controlled for maximum production.

In this study, we demonstrated an efficient method of converting acetate to tyrosine via precise tuning of the glyoxylate cycle in *Escherichia coli*. To increase the carbon flux, acetate assimilation and gluconeogenesis pathways were activated by overexpression of *acs* (encoding acetyl-CoA synthetase) and *pck* (encoding PEP carboxykinase) in the SCK1 strain, a previously constructed tyrosine-producing strain [17]. Thereafter, the activity of the glyoxylate cycle was precisely controlled by varying the expression of *aceA,* a key gene encoding isocitrate lyase. The engineered strain with optimal glyoxylate cycle activity showed significantly enhanced tyrosine production with yield. This study is the first attempt at tyrosine production from acetate and indicates the potential of acetate as a feedstock for producing various chemicals similar to that shown in previous studies [11–13].

## Results and discussion

### Evaluation of SCK1 for tyrosine production from acetate

Previously, we had reconstructed the tyrosine production pathway in SCK1, an *E. coli* K-12 W3110 strain using the synthetic promoters and UTRs (Table 1) [17]. Although we further overexpressed *ppsA* due to its crucial gluconeogenic activity during glucose utilization [17], the SCK1 strain without additional engineering was chosen as this gene is natively up-regulated during acetate assimilation [32, 35].

**Table 1:** Bacterial strains and plasmids used in this study

Name	Description	Source
Strains		
*E. coli* Mach1-T1^R^	Cloning host	Invitrogen
SCK1	W3110 Δ*tyrR aroG*:: P_BBa_J23100_-synUTR_aroG_-*aroG*^*fbr*^ *tyrA*:: P_BBa_J23100_-synUTR_tyrA_-*tyrA*^*fbr*^ P_aroABCDELtyrB_-UTR_aroABCDELtyrB_:: P_BBa_J23100_-synUTR_aroABCDELtyrB_	[17]
SCKE	SCK1/pACYCduet-1	This study
SCKA	SCK1/pACA	This study
SCKP	SCK1/pACP	This study
SCKAP	SCK1/pACAP	This study
SCKDIAP	SCK1 Δ*iclR::*FRT-Km^R^-FRT/pACAP	
SCKAPG1	SCK1/pACAPG1	This study
SCKAPG2	SCK1/pACAPG2	This study
SCKAPG3	SCK1/pACAPG3	This study
SCKAPG4	SCK1/pACAPG4	This study
SCKAPG5	SCK1/pACAPG5	This study
Plasmids		
pKD46	Red recombinase expression vector, Amp^R^	[40]
pM_FKF	PCR template for FRT-Kan^R^-FRT, pMB1 ori, Amp^R^, Km^R^	[12]
pACYCduet-1	p15A ori, Cm^R^, *E. coli* expression vector	Novagen
pACA	p15A ori, Cm^R^, P_BBa_J23100_-synUTR_acs_-*acs*-Ter_BBa_B1006_	This study
pACP	p15A ori, Cm^R^, P_BBa_J23100_-synUTR_pck_-*pck*-Ter_BBa_B1006_	This study
pACAP	p15A ori, Cm^R^, P_BBa_J23100_-synUTR_acs_-*acs*-Ter_BBa_B1006_-P_BBa_J23100_-synUTR_pck_-*pck*-Ter_BBa_B1006_	This study
pACAPG1	p15A ori, Cm^R^, P_BBa_J23100_-synUTR_acs_-*acs*-Ter_BBa_B1006_-P_BBa_J23100_-synUTR_pck_-*pck*-Ter_BBa_B1006_-P_BBa_J23104_-synUTR_aceA_-*aceA*-Ter_BBa_B1006_	This study
pACAPG2	p15A ori, Cm^R^, P_BBa_J23100_-synUTR_acs_-*acs*-Ter_BBa_B1006_-P_BBa_J23100_-synUTR_pck_-*pck*-Ter_BBa_B1006_-P_BBa_J23118_-synUTR_aceA_-*aceA*- Ter_BBa_B1006_	This study
pACAPG3	p15A ori, Cm^R^, P_BBa_J23100_-synUTR_acs_-*acs*-Ter_BBa_B1006_-P_BBa_J23100_-synUTR_pck_-*pck*-Ter_BBa_B1006_-P_BBa_J23116_-synUTR_aceA_-*aceA*-Ter_BBa_B1006_	This study
pACAPG4	p15A ori, Cm^R^, P_BBa_J23100_-synUTR_acs_-*acs*-Ter_BBa_B1006_-P_BBa_J23100_-synUTR_pck_-*pck*-Ter_BBa_B1006_-P_BBa_J23109_-synUTR_aceA_-*aceA*- Ter_BBa_B1006_	This study
pACAPG5	p15A ori, Cm^R^, P_BBa_J23100_-synUTR_acs_-*acs*-Ter_BBa_B1006_-P_BBa_J23100_-synUTR_pck_-*pck*-Ter_BBa_B1006_-P_BBa_J23100_-synUTR_aceA_-*aceA*-Ter_BBa_B1006_	This study

*Amp* ampicillin, *Cm* chloramphenicol, *Km* kanamycin; *R* resistance

**Fig. 2:**
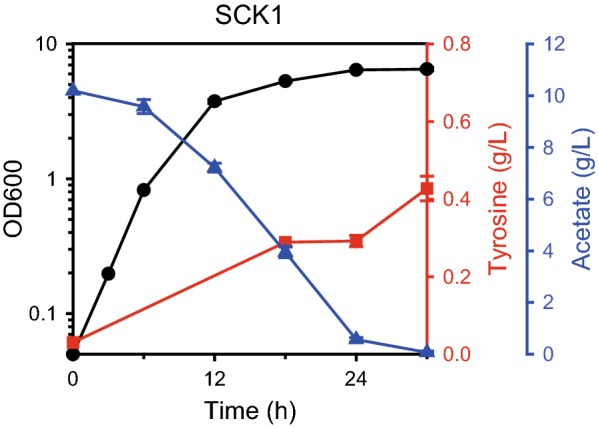
Fermentation profile of the SCK1 strain. The left *y*-axis shows OD_600_ and the right *y*-axis indicates concentration of the accumulated tyrosine; the *y*-offset indicates the concentration of the remaining acetate. The *x*-axis denotes time. Symbols: black circle, cell biomass (OD_600_); red squares, tyrosine; blue diamonds, acetate. Error bars indicate the standard deviation from three independent cultures

Initially, its ability for tyrosine production was investigated in modified minimal medium with 10 g/L acetate as the sole carbon source (Fig. 2). Although acetate is known to inhibit cellular growth [12], the SCK1 strain showed moderate cell growth (0.47/h) compared to the growth rate of the wild-type W3110 strain (0.45/h), even with overexpression of genes related to tyrosine production. After 30 h of fermentation, the strain successfully produced 0.43 g/L tyrosine by consuming all provided acetate. The yield was 11% of the theoretical maximum yield (0.375 g tyrosine/g acetate; see “Methods” section and Additional file 1 for the calculation).

### Effect of *acs* and *pck* overexpression on tyrosine production

**Fig. 3:**
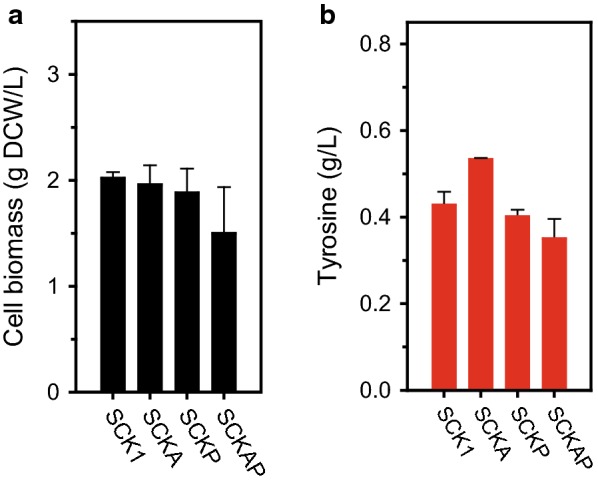
Effect of the expression of *acs* and *pck*. Cell biomass (**a**) and tyrosine production (**b**) after 30 h cultivation. Error bars indicate the standard deviation from three independent cultures

The precursors, PEP and E-4-P, should be readily available for enhancing tyrosine production [17]. However, the acetate utilization rate is known to be slower than the rate of glucose utilization [32] and it potentially decreases tyrosine production. To accelerate acetate assimilation, the acetate uptake pathway was amplified by overexpression of *acs* [11, 12, 36]. The synthetic expression cassette for *acs* was constructed with the strong constitutive promoter (P_J23100_) and synthetic 5′ UTR (Additional file 1: Table S3) in a low copy plasmid (pACYCduet-1), and the final plasmid was named pACA. Then, the SCKA strain harboring the resulting pACA was cultivated to evaluate the effect of *acs* overexpression (Fig. 3a, b). The introduction of pACYC yielded 1.4-fold enhanced *acs* expression indicating the successful overexpression (Additional file 1: Figure S1a). Unlike our previous observations [12, 13], overexpression of *acs* slightly reduced specific acetate consumption rate (a 1.2-fold, 0.18 g/g DCW/h) (Fig. 3a, b). Nevertheless, tyrosine production was increased by a 1.2-fold to 0.53 g/L. Furthermore, the yield was notably increased (a 1.6-fold), suggesting efficient utilization of acetate.

Next, we investigated the effect of overexpressing *pck*; this gene was selected as it is known as a major reaction for PEP supplementation [32]. We constructed the SCKP strain (harboring a synthetic *pck* overexpression cassette in the pACP plasmid) in a similar manner that used for generating the SCKA strain. 1.4-fold increased level of *pck* expression was observed in SCKP strain (Additional file 1: Figure S1b). In this case, the SCKP strain showed a marginal reduction in cell biomass (a 1.1-fold, 1.9 g DCW/L, Fig. 3a) in addition to more reduced specific acetate consumption rate (a 1.7-fold, 0.13 g/g DCW/h, Table 2); these negative results were probably due to the reduced TCA cycle activity with a loss of oxaloacetate (Fig. 1a, b). Even with the lowered acetate consumption, tyrosine production was maintained in a similar level (0.40 g/L, Fig. 3b) with slightly higher yield (a 1.2-fold increase). This result indicates that carbon flux was directed to tyrosine synthesis by overexpression of *pck*.

**Table 2:** Fermentation profile of engineered *E. coli*

Strain	Dry cell weight (g/L)	Specific acetate consumption rate (g/g DCW/h)	Acetate consumption (g/L)	Tyrosine (g/L)	Percentage yield (%)^a^
SCK1	2.0 ± 0.1	0.22 ± 0.01	9.93	0.43 ± 0.03	11 ± 1
SCKA	2.0 ± 0.2	0.18 ± 0.00	8.33	0.53 ± 0.00	17 ± 0
SCKP	1.9 ± 0.2	0.13 ± 0.03	8.38	0.40 ± 0.02	13 ± 1
SCKAP	1.5 ± 0.4	0.14 ± 0.04	5.44	0.35 ± 0.05	17 ± 2
SCKAPG1	2.8 ± 0.1	0.21 ± 0.03	10.00	0.48 ± 0.02	9 ± 1
SCKAPG2	2.8 ± 0.2	0.21 ± 0.02	10.00	0.42 ± 0.10	12 ± 3
SCKAPG3	2.9 ± 0.1	0.19 ± 0.02	10.00	0.47 ± 0.01	12 ± 0
SCKAPG4	2.8 ± 0.2	0.21 ± 0.02	10.00	0.70 ± 0.11	20 ± 3
SCKAPG5	2.6 ± 0.1	0.20 ± 0.01	10.00	0.49 ± 0.04	12 ± 1.0
SCKDIAP	1.0 ± 0.2	0.05 ± 0.00	4.79	0.33 ± 0.01	18 ± 1

^a^Percentage yield indicates the ratio of actual yield to theoretical maximum yield expressed in percentage (%)

The effect of combined strategies was also investigated. Both synthetic expression cassettes were integrated into a single plasmid to obtain pACAP. Higher gene expression of each gene was maintained in the SCKAP strain (SCK1 harboring the pACAP plasmid, Additional file 1: Figure S1a, b). Similar to the observations above, the SCKAP strain showed low cell biomass (a 1.3-fold decrease, 1.5 g DCW/L) and specific acetate consumption (a 1.6-fold decrease, 0.14 g/g DCW/L). Consequently, 1.2-fold decreased tyrosine production was also observed (0.35 g/L). However, the yield and intracellular PEP level were improved (a 1.7-fold increase and 2.3-fold, respectively) compared to SCK1 strain (Additional file 1: Figure S2) which implies that tyrosine production pathway was amplified. To enhance tyrosine production, we believed that reduced biomass formation and acetate consumption should be recovered by pathway optimization.

### Tuning the glyoxylate cycle by varying the expression of *aceA* for improved tyrosine production

We attempted to additionally activate glyoxylate cycle to enhance the PEP availability. However, it should be elaborately controlled to maintain sufficient generation of ATP and NADH via the TCA cycle (Fig. 1b) [34]. The precise flux control could be implemented by varying the transcriptional efficiency of *aceA* encoding the first enzyme of glyoxylate cycle [12, 34]. The *aceA* expression cassettes, with a series of constitutive promoters (J231 series), were integrated into the pACAP plasmid, resulting in the pACAPG1-5 plasmids (Table 1).

**Fig. 4:**
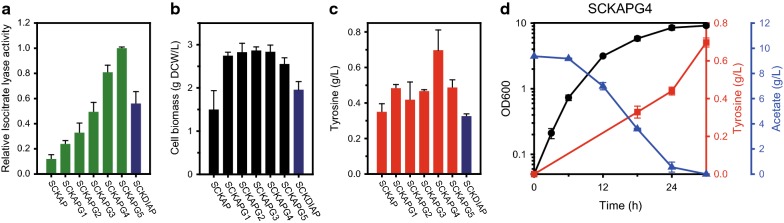
Effect of glyoxylate cycle activation. Comparison of normalized specific isocitrate lyase activity measured at 12 h (**a**), and cell biomass (**b**) and tyrosine production (**c**) after 30 h cultivation, and the fermentation profile of the SCKAPG4 strain (**d**). The left *y*-axis shows OD_600_ and the right y-axis indicates the concentration of accumulated tyrosine; the *y*-offset indicates the concentration of the remaining acetate. The *x*-axis denotes time. Symbols: black circle, cell biomass (OD_600_); red squares, tyrosine; blue diamonds, acetate. Error bars indicate the standard deviation from three independent cultures

The SCKAPG1-5 strains (SCK1 harboring pACAPG1-5, respectively, Table 1) showed successfully diversified AceA activity of up to 8.3-fold (Fig. 4a). As shown in Fig. 4b, the activation of the glyoxylate cycle may lead to an increase in cell biomass, as the activated anaplerosis recovered cell biomass formation. In particular, the slight difference in AceA activity between strains SCKAP and SCKAPG1 was sufficient for stimulating cell growth (a 1.8-fold increase, 2.8 g DCW/L), as no notable increase in cell biomass was observed with higher AceA activity. In contrast, tyrosine production was critically affected by AceA activity, and maximum tyrosine production was observed in the SCKAPG4 strain with the second highest AceA activity (Fig. 4c, d). In addition, the SCKAPG5 strain with maximum AceA activity showed lower tyrosine production than the SCKAPG4 strain (Fig. 4c). These results suggest that the optimal flux redistribution around the glyoxylate cycle and TCA cycle achieved by regulating *aceA* expression is critical for tyrosine production. The SCKAPG4 strain showed 0.70 g/L tyrosine production, which is 2.0-fold higher than that of the SCKAP strain and 1.6-fold higher than that of the SCK1 strain. The acetate consumption rate was substantially recovered (Table 2) and the yield was also significantly increased to 20% of the theoretical maximum (Table 2) [17]. Furthermore, a 5.4-fold increased intracellular PEP level was observed compared to SCK1 strain as we expected (Additional file 1: Figure S2). Collectively, these results indicate that carbon flux distribution can be successfully used for efficient tyrosine production from acetate.

### Comparison with *iclR* deletion approach

Finally, we compared our strategy with the conventional strategy for activation of the glyoxylate cycle, the deletion of *iclR* [37]. The SCKDIAP strain (SCKAP with *iclR* deletion) showed significantly increased AceA activity (Fig. 4a); however, severely reduced cell biomass (a 1.4-fold decrease, 1.5 g DCW/L, Fig. 4b) and tyrosine production (a 1.1-fold decrease, 0.33 g/L, Fig. 4c) were observed compared to the SCKAP strain. These parameters are lower even compared to the SCKAPG3 strain with similar AceA activity. This can be explained by the regulation of *iclR*; activation of *aceK,* encoding isocitrate dehydrogenase kinase/phosphatase, blocks the oxidative flux in TCA cycle [37], resulting in severe imbalance in tyrosine production. Overall, these results indicate that our precise tuning strategy is more efficient and has considerable potential for chemical production [12, 34].

## Conclusions

In this study, we demonstrated a method for efficient production of tyrosine from acetate. Initially, the SCK1 strain with amplification of the PEP to tyrosine synthetic pathway was utilized. To maximize carbon flux toward tyrosine, the acetate uptake and gluconeogenic pathways were additionally amplified. Although the overexpression of the linear pathway itself could not improve tyrosine production due to metabolic imbalance, rational pathway optimization could be achieved by precise regulation of the glyoxylate cycle. Finally, the engineered strain, SCKAPG4, produced 0.70 g/L tyrosine (a 1.6-fold increase compared to parental strain) with 20% of the theoretical maximum yield. Although the achieved titer is lower than previous studies (Additional file 1: Table S1) and the engineered strains still produced more tyrosine using glucose (1.23 g/L, Additional file 1: Fig. S3), the results are still promising to show the potential of acetate as an alternative feedstock. Future studies including acetate toxicity, related stress response, process optimization, and balancing between PEP and E-4-P would enhance the production further. In addition, these strategies will be utilized for producing other gluconeogenesis- and TCA-derived chemicals.

## Methods

### Reagents and oligonucleotides

Plasmid DNA and genomic DNA were extracted using the GeneAll^R^ Plasmid SV kit and GeneAll^R^ Exgene™ Cell SV kit (GeneAll, Seoul, Korea), respectively. Q5^R^ High-Fidelity DNA polymerase, T4 DNA ligase, and restriction endonucleases were purchased from New England Biolabs (Ipswich, MA, USA). The oligonucleotides were synthesized by Cosmogenetech (Seoul, Korea) and are listed in Additional file 1: Table S2. The GeneAll^R^ Expin™ Gel SV and GeneAll^R^ Expin™ CleanUp SV kits were used for purification of DNA. Other reagents for cell culture and enzyme assay were purchased from Sigma-Aldrich (St. Louis, MO, USA).

### Construction of strains and plasmids

All bacterial strains and plasmids used in this study are listed in Table 1. Routine cloning was performed using *E. coli* Mach1-T1^R^ (Thermo scientific, Waltham, MA, USA) as the host. For tyrosine production, the SCK1 strain with re-designed tyrosine synthetic pathway was used [17]. The genes (*acs, pck,* and *aceA*) were obtained from the genomic DNA of *E. coli* strain W3110. The synthetic promoter (J231 promoter series) and terminator (BBa_B1006) were used from the Registry of Standard Biological Parts (http://parts.igem.org) for expression of the gene in a quantitative manner. The synthetic 5′-UTRs were designed using the UTR designer (http://sbi.postech.ac.kr/utr_designer) for efficient translation and are listed in Additional file 1: Table S3 [38].

The Golden Gate cloning strategy [39] with the *BsaI* endonuclease was used for plasmid construction. To prepare pACA and pACP, a vector fragment was amplified with primers pACYC_F/pACYC_ R and plasmid pACYCduet-1 as the template. *acs* and *pck* fragments were amplified with primer pairs acs_F/acs_R and pck_F/pck_R, respectively. Then, the resulting fragments were digested with *BsaI* and assembled. Similarly, a vector fragment was amplified with pACYC_multi_F/pACYC_multi_R primers and pACA as the template for constructing pACAP and pACAPG1-5. The vector fragment was assembled with fragments amplified with pck_multi_F/pck_multi_R and aceA_F1-5/aceA_R, respectively. For chromosomal deletion of *iclR*, a FRT-Kan^R^-FRT fragment was prepared with the iclR_del_F/iclR_del_R and pM_FKF as the template. Thereafter, the fragment was transformed into the SCK1 strain according to the lambda-red recombination method using pKD46 [40].

### Culture medium and culture conditions

Cells were cultivated in modified minimal medium containing 0.5 g/L MgSO_4_∙7H_2_O, 2.0 g/L NH_4_Cl, 1.0 g/L NaCl, 2.0 g/L yeast extract, and 100 mM potassium phosphate buffer (pH 7.0). Neutralized acetate with sodium hydroxide (pH 7.0) or glucose was used as the carbon source. To maintain plasmids, appropriate concentrations of antibiotics were added to the medium (50 µg/mL streptomycin, 50 µg/mL kanamycin, and 100 µg/mL ampicillin).

Cell culture was conducted in 300-mL Erlenmeyer flasks containing 25 mL modified minimal medium. For seed culture, a single colony was inoculated in a 15-mL test tube containing 3 mL medium with 5 g/L acetate. After 12 h, the seed culture was inoculated in a fresh 10 g/L acetate medium and cultured at 37 °C with agitation at 200 rpm to an optical density at 600 nm (OD_600_) of 0.05. The pH was adjusted to 6.5–6.8 with a 5 M HCl solution. The experiments were conducted in biological triplicate. Culture samples were periodically taken and stored at − 80 °C until analysis.

### Quantification of cell biomass and metabolites in medium

Cell biomass was measured using a UV-1700 spectrophotometer (Shimadzu, Kyoto, Japan) at a wavelength of 600 nm, and one OD_600_ unit was converted to 0.31 g/L of dry cell weight (DCW) [12]. The pH was measured using an Orion™ 8103BN ROSS™ pH meter (Thermo Scientific).

High-performance liquid chromatography (HPLC) was used to quantify the metabolites. The samples were centrifuged and passed through a 0.22-μm syringe filter. The filtered samples were analyzed using an UltiMate™ 3000 analytical HPLC system (Dionex, Sunnyvale, CA, USA). Acetate consumption was determined using an Aminex HPX-87H column (Bio-Rad Laboratories, Richmond, CA, USA) at a flow rate of 0.6 mL/min at 14 °C using 5 mM H_2_SO_4_ as the mobile phase. The refractive index (RI) was monitored using a Shodex RI-101 detector (Shodex, Klokkerfaldet, Denmark). A pre-column o-phthalaldehyde derivatization method, coupled with reverse-phase liquid column chromatography, was used for detecting tyrosine production (Acclaim 120 C18; Dionex, Sunnyvale, CA, USA). Derivatized tyrosine was eluted at a flow rate of 1.5 mL/min with a gradient of acetonitrile, methanol, and water (45:45:10% of [v/v]) and 50 mM sodium acetate buffer. The ultraviolet–visible (UV–Vis) diode array detector was used for signal detection.

### Quantification of intracellular PEP

Relative intracellular metabolite was analyzed using GC–MS system. For metabolite extraction, cells (5 × 10^9^) were collected by filtration using a mixed cellulose ester membrane disk filter of pore size 0.22 μm (Millipore, Bedford, MA). Then, pre-chilled isotonic solution (9 g/L NaCl in water) was filtrated to wash media components. After then, the filter was immersed in 4 mL of chloroform/methanol/water (32:60:8, v/v/v) solution and vortexed. The solution was centrifuged at 8000 rpm for 10 min at 0 °C and 0.5 mL of aqueous phase were transferred to 1.5 mL tube. DL-norvaline (Sigma-Aldrich) as an internal standard was added. Finally, the solution was fully dried using a vacuum dryer (Hanil Science Industrial Co., Incheon, Korea) and kept on − 80 °C until analyzed [41]. Samples were dissolved in 50 μL of MOX solution (pyridine containing 20 mg/mL methoxyamine hydrochloride) and incubated at 70 °C for 50 min on a heating block. Then, 80 μL of BSTFA + 10% TMCS (Sigma Aldrich) was added and incubated at 70 °C for 50 min. After then, the samples were centrifuged at 14,000 rpm for 10 min to pellet any undissolved residue, and the supernatant was transferred to a new glass insert for GC–MS injection.

Sample analysis was performed on a GC–MS system, GC 7890 coupled to an MSD 5977 (Agilent Technologies, Inc., Santa Clara, CA, USA) equipped with a HP-5MS capillary column (30 cm × 0.25 mm i.d. × 0.25 mm; Agilent J&W Scientific). The injection volume was 1 μL and all samples were run in 1:10 split mode with an inlet temperature of 270 °C. Helium flow rate was set to 1 mL/min. The MS source temperature was maintained at 230 °C, and the MS quad temperature was held constant at 150 °C with electron energy of 70 eV. The oven temperature profile was 80 °C for 5 min; 10 °C/min to 300 °C, and held at 300 °C for 5 min. The selected ions monitored were 144 m/z for dl-norvaline, 369 m/z for PEP [42].

### Measurement of gene transcripts

To measure the amount of *acs* and *pck* transcripts, total RNAs were extracted using Ribospin™ and Riboclear™ plus (GeneAll) from the cells cultured for 12 h. Complementary DNA was synthesized using SuperScript III Reverse Transcriptase (Invitrogen, Carlsbad, CA) and it was measured by quantitative PCR using a StepOnePlus Real-time PCR system (Applied Biosystems, Foster City, CA). For the assay, TOPreal™ qPCR 2X PreMIX (Enzynomics, Daejeon, Korea) was utilized. The primers RT_acs_F/RT_acs_R and RT_pck_F/RT_pck_R were used for amplification of each gene transcripts. The amounts of the transcripts were quantified using the comparative C_T_ method [43] with amplified *cysG* transcript as an internal standard with RT-cysG_F/RT_cysG_R. The experiments were conducted in technical triplicates of one representative sample.

### Measurement of isocitrate lyase activity

To measure the enzymatic activity of isocitrate lyase, samples were collected at 12 h and analyzed as reported previously [34]. Specifically, the cells were cultured for 12 h and centrifuged to obtain the amount corresponding to 1 mL suspension with OD_600_ of 1.5. The cells were lysed by addition of the Bug Buster master mix (EDBioscience, San Diego, CA, USA). Thereafter, the cell lysate was added to a reaction cocktail containing 250 mM potassium phosphate, 100 mM MgCl_2_, 100 mM phenylhydrazine, 100 mM cysteine, and 100 mM isocitrate. The absorbance at 324 nm was monitored using the VICTOR3 1420 multilevel plate reader (PerkinElmer, Waltham, MA, USA) at room temperature. The measurement was performed in technical triplicates.

### Calculation of the theoretical maximum yield

The reaction for producing tyrosine from acetate involves more than 30 enzymatic steps, with entry and exit of diverse compounds and energy. For detailed information, please refer to Stoichiometry for calculating theoretical maximum yield in Additional file 1. Initially, 8 mol of acetate can be converted to 4 mol of oxaloacetate via the glyoxylate cycle and TCA cycle (Additional file 1: Equations 1–9). Then, 4 mol of oxaloacetate is converted to 4 mol of PEP, with the release of 4 mol CO_2_ (Additional file 1: Equations 10, 11). Among them, 2 mol of PEP are condensed to 1 mol of E-4-P via gluconeogenesis and the pentose phosphate pathway (Additional file 1: Equations 12–25). The remaining 2 mol of PEP are condensed with the resulting E-4-P to synthesize 1 mol of tyrosine (Additional file 1: Equations 26–37). Overall, 21 mol of ATP are consumed during the conversion of 8 mol of acetate to 1 mol of tyrosine (Additional file 1: Equation 38). The considerable energy consumption during the conversion can be compensated by oxidation of the generated reducing cofactors. Consequently, the theoretical maximum yield is 0.125 mol of tyrosine per mol of acetate (0.375 g tyrosine/g acetate). On the other hand, the theoretical maximum yield of production from glucose was calculated (Additional file 1: equation 39–51) to be 0.5 mol of tyrosine per mol of glucose (0.5 g tyrosine/g glucose).

## Additional file

**Additional file 1.** Previous studies for microbial tyrosine production and nucleotide sequence used in this studies were summarized. In addition, additional results including stoichiometry and intracellular PEP concentration are presented to help understand the our engineering strategy.
