# Supplementary material for: Precise tuning of the glyoxylate cycle in Escherichia coli for efficient tyrosine production from acetate
Source: Microb Cell Fact. 2019 Mar 19;18:57. doi: 10.1186/s12934-019-1106-0 (PMC6423740; doi:10.1186/s12934-019-1106-0)
Supplement: Supplementary file 1 — Additional file 1. Previous studies for microbial tyrosine production and nucleotide sequence used in this studies were summarized. In addition, additional results including stoichiometry and intracellular PEP concentration are presented to help understand the our engineering strategy. [file 12934_2019_1106_MOESM1_ESM.docx]

**Additional information for**

**Precise tuning of the glyoxylate cycle in *Escherichia coli* for efficient tyrosine production from acetate**

Minji Jo^†^, Myung Hyun Noh^†^, Hyun Gyu Lim^†^, Chae Won Kang, Dae-Kyun Im,
Min-Kyu Oh, and Gyoo Yeol Jung^*^

^†^These authors contributed equally to this work.

*To whom correspondence should be addressed.
E-mail :

[mjijo@potech.ac.kr](mailto:mjijo@potech.ac.kr) (M. J.)

[mh2541@postech.ac.kr](mailto:mh2541@postech.ac.kr) (M. H. N.)

[airbo2@postech.ac.kr](mailto:airbo2@postech.ac.kr) (H. G. L.)

[codnjs6897@postech.ac.kr](mailto:codnjs6897@postech.ac.kr) (C. W. K.)

[meganti@korea.ac.kr](mailto:meganti@korea.ac.kr) (D-K. I.)

[mkoh@korea.ac.kr](mailto:mkoh@korea.ac.kr) (M-K. O.)

[gyjung@postech.ac.kr](mailto:gyjung@postech.ac.kr) (G. Y. J.)

**Table of Contents:**

**Supplementary Table S1 – S3**

**Supplementary Figures S1 – S3**

**Stoichiometry for calculating theoretical maximum yield**

**Supplementary Tables
Table S1. Previous studies for microbial tyrosine production.**

| **Carbon source** | **Supplement** | **Strategy** | **Titer**  **(time)** | **Yield^a^** | **Fermentation mode** | | **Reference** | | |
| --- | --- | --- | --- | --- | --- | --- | --- | --- | --- |
| Sucrose | Yeast extract  (5 g/L) | *E. coli* NST 37 / ΔP_trc_*-tyrA* Δ*pheLA* | 0.08 g/L  (45 h) | 0.01 | Fed-batch | | [1] | |  |
| Glucose | Tryptophan  (10 g/L)  Phenylalanine  (10 g/L) |  | 0.18 g/L  (42 h) | 0.09 | Fed-batch | |  |  |  |
| Glucose | - | *E. coli* W3110 Δ*ptsI* Δ*ptsH* Δ*lacI* Δ*crr lacZ*::*loxP* P*_galP_*::P*_trc_* Δ*tyrR* / P_lacUV_*_5_-aroG^fbr^-tktA* / P_trc_-*tyrC-pheA_CM_* | 0.15 g/L  (-) | 0.36 | Flask | | [2] | |  |
| Glucose | - | *E. coli* K-12 Δ*pheA* Δ*tyrR* Δ*ygdT* / P_lac_-*tyrA^fbr^-aroG^fbr^* | 0.59 g/L  (24 h) | 0.12 | Flask | | [3] | |  |
| Glucose | - | *E. coli* K-12 Δ*tyrR* / P_LtetO-1_*-aroG^fbr^-tyrA^fbr^-ppsA-tktA* / P_LtetO-1_-*aroK-ydiB* | 0.70 g/L  (16 h) | 0.14 | Flask | | [4] | |  |
| Glucose | - | *E. coli* MG1655 / P_lac-uv_*-aroE-aroD-aroB^op^* P_LtetO-1-_*aroG^*^-ppsA-tktA* / P_lac-uv-_*tyrB- tyrA^fbr^-aroC*T1-P_trc_-*aroA*-*aroL* | 2.2 g/L  (48 h) | 0.44 | Flask | | | [5] |  |
| Glucose | Yeast extract  (10 g/L) | *E. coli* PB12 derived from JM101 / P_cyc_-*aroG^fbr^* / P_trc-_*tyrC-pheA*_CM_ | 3.0 g/L (52 h) | 0.06 | Bioreactor | | | [6] |  |
| Glucose | Yeast extract  (3 g/L)  Citrate  (0.85 g/L) | *E. coli* W3110 Δ*tyrR aroG*::P_BBa_J23100_-synUTR_aroG_-*aroG^fbr^ tyrA*::P_BBa_J23100_-synUTR_tyrA_-*tyrA^fbr^* P_aroABCDELtyrB_-UTR_aroABCDELtyrB_::P_BBa_J23100_-synUTR_aroABCDELtyrB_ P_ppsA-_UTR_ppsA_::P_BBa J23100-_synUTR_ppsA_ | 3.0 g/L  (48 h) | 0.10 | Batch | | | [7] |  |
| Glucose | Yeast extract  (2 g/L) | *E. coli* W3110 Δ*tyrR* Δ*pheA* Δ*tyrA* / pMGL-*aroL-aroG4* / P_lacI_*-tyrA^fbr^* | 6.3 g/L  (28 h) | 0.16 | | Flask | | [8] |  |
| Glucose | Citrate  (0.2 g/L) | *E. coli* K-12 Δ*tyrR* / P_tet_-*tyrA^fbr^-aroG^fbr^-ppsA-tktA* | 9.7 g/L  (-) | 0.10 | | Batch | | [9] |  |
| Glucose | - | *E. coli* K-12 Δ*tyrR* / P_LtetO-1_*-aroG^fbr^-tyrA^fbr^-ppsA-tktA*-*rpoA14** | 14 g/L  (88 h) | 0.12 | | Fed-batch | | [10] |  |
| Glucose | Phenylalanine  (1 g/L)  Yeast extract  (2 g/L) | *E. coli* L-Phe producer generated by random mutagenesis Δ*pheA* Δ*pheL* P_tyrA_:: P_trc_ | 55 g/L  (48 h) | 0.3 | | Fed-batch | | [11] |  |

^a^Yield (g sugar/g tyrosine)

**Table S2.** Oligonucleotides used in this study

| **Name** | **Sequence (5′−3′)^a,b^** |  |
| --- | --- | --- |
| pACYC_F | ttgctgggtctcgttcttcttgagatcgttttggtctgcg | |
| pACYC_R | ttgctgggtctcgcaataaaaaaattacgccccgccctg | |
| acs_F | ttgctgggtctcgatgacggctagctcagtcctaggtacagtgctagcAAAATCAGCGCCCAAGGAGTCACCGatgagccaaattcacaaacacacc | |
| acs_R | ttgctgggtctcgagaaaaaaaaccccgccctgtcaggggcggggtttttttttttacgatggcatcgcgatagc | |
| pck_F | ttgctgggtctcgattgacggctagctcagtcctaggtacagtgctagcCAAAACTACAAAAGGAGGATCAAAAatgcgcgttaacaatggtttg | |
| pck_R | ttgctgggtctcgagaaaaaaaaccccgccctgtcaggggcggggtttttttttttacagtttcggaccagccgctac | |
| pACYC_multi_F | cattccggtctcgtcttcttgagatcgttttggtctgcg | |
| pACYC_multi_R | cattccggtctcgcaaaaaaaaaaccccgccctgtcag | |
| pck_multi_F | cattccggtctcgtttgacggctagctcagtcctagg | |
| pck_multi_R | cattccggtctccaagaaaaaaaaccccgccctgtcag | |
| aceA_F1 | cattccggtctcgtcttttgacagctagctcagtcctaggtattgtgctagcGTGATCTAGAAAAGGAGCATCCGTA atgaaaacccgtacacaacaaat | |
| aceA_F2 | cattccggtctcgtcttttgacggctagctcagtcctaggtattgtgctagcGTGATCTAGAAAAGGAGCATCCGTA atgaaaacccgtacacaacaaat | |
| aceA_F3 | cattccggtctcgtcttttgacagctagctcagtcctagggactatgctagcGTGATCTAGAAAAGGAGCATCCGTA atgaaaacccgtacacaacaaat | |
| aceA_F4 | cattccggtctcgtctttttacagctagctcagtcctagggactgtgctagcGTGATCTAGAAAAGGAGCATCCGTA atgaaaacccgtacacaacaaat | |
| aceA_F5 | cattccggtctcgtcttttgacggctagctcagtcctaggtacagtgctagcGTGATCTAGAAAAGGAGCATCCGTA atgaaaacccgtacacaacaaat | |
| aceA_R | cattccggtctccaagaaaaaaaaccccgccctgtcaggggcggggtttttttttttagaactgcgattcttcagtgg | |
| iclR_del_F | atggtcgcacccattcccgcgaaacgcggcagaaaacccgccgttgccacccgcatgaccgcgcgatgc | |
| iclR_del_B | tcagcgcattccaccgtacgccagcgtcacttccttcgccgctttaatcacgcgacgacaggcacatgcg | |
| RT_acs_F | actgggtgcgtaaagaga | |
| RT_acs_R | agaatacggcgcataattt | |
| RT_pck_F | gtagacacgaagattctcgat | |
| RT_pck_R | tcgaagttgtcgataaaca | |
| RT_cysG_F | gttctatatggggttgaatc | |
| RT_cysG_R | gtaccgttttcgacaatt | |

^a^Underlined letters indicate the restriction enzyme site (*BsaI*).
^b^Uppercase letters in the sequence represent the synthetically designed 5′ UTR.

**Table S3.** Designed 5′ UTR sequences and their predicted expression levels in this study

| **Gene** | | **5′ UTR sequence (5′-3′)** | **Predicted**  **expression level^a^**  **(a.u.)** |
| --- | --- | --- | --- |
| *acs* | AAAATCAGCGCCCAAGGAGTCACCG | 1,074,836.02 | |
| *pck* | CAAAACTACAAAAGGAGGATCAAAA | 9,348,010.52 | |
| *aceA* | GTGATCTAGAAAAGGAGCATCCGTA | 1085302.27 | |

^a^Arbitrary unit of the expression [12]

**Supplementary Figures**

**Fig. S1.** The relative gene expression level of *acs* (a) and *pck* (b). Each gene expression level was normalized by the value of the SCK1 strain. Error bars indicate the standard deviations from three technical replicates.


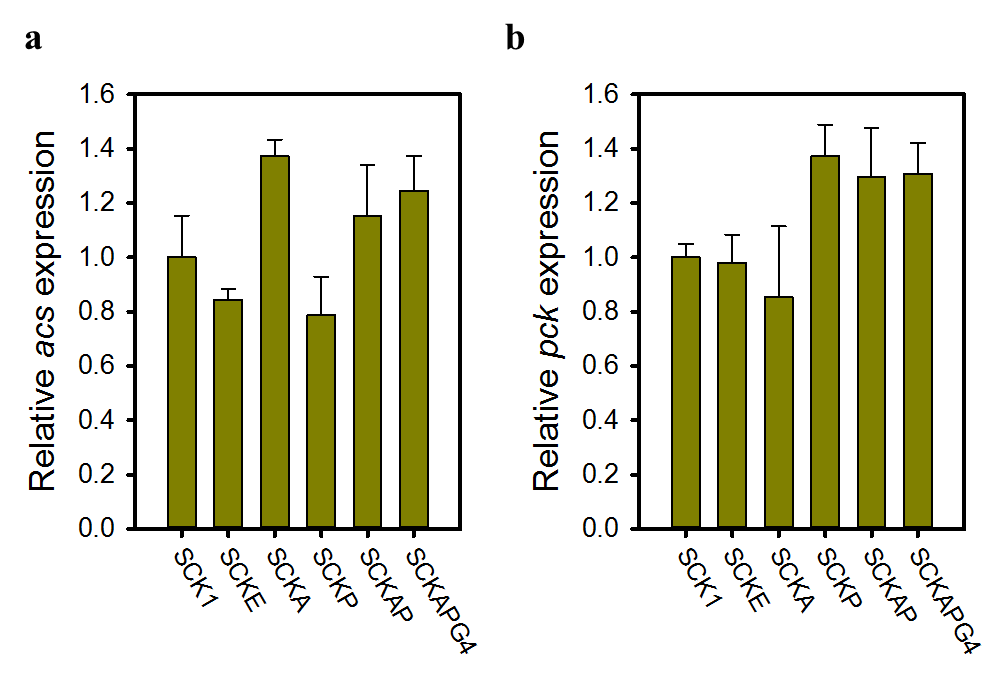


**Fig. S2.** The relative amount of intracellular PEP of the engineered strains. Each intensity from GC-MS analysis was normalized by the value of the SCK1 strain. Error bars indicate the standard deviations from three replicates.

**Fig. S3.** Fermentation profile of the engineered strains with glucose as a carbon source. Cell biomass (a), tyrosine production (b) and percentage yield of theoretical maximum yield (c) after 18 h cultivation. Error bars indicate the standard deviation from three independent cultures.


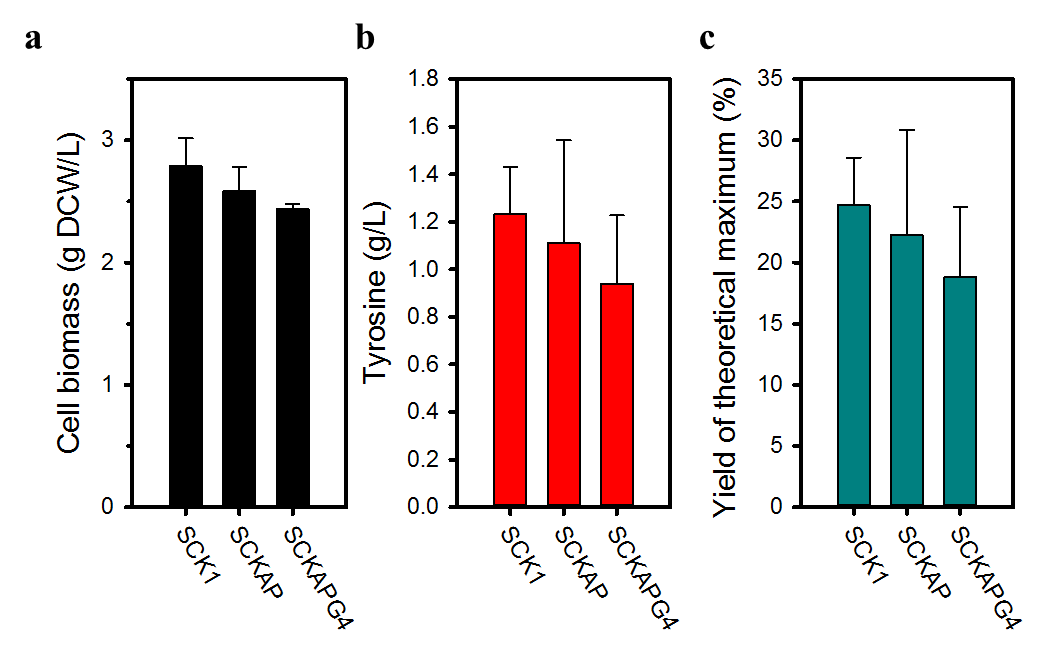


**Stoichiometry for calculating theoretical maximum yield**

***From 8 mol of acetate to 4 mol of PEP***8 Acetate + 16 ATP + 8 CoA → 8 Acetyl-CoA + 16 ADP + 16 P_i_ (equation 1)
4 Acetyl-CoA + 4 Oxaloacetate + 4 H_2_O → 4 Citrate + 4 CoA (equation 2)
4 Citrate → 4 *cis*-Aconitate + 4 H_2_O    (equation 3)
4 *cis*-Aconitate + 4 H_2_O → 4 Isocitrate (equation 4)

4 Isocitrate → 4 Succinate + 4 Glyoxylate (equation 5)

4 Glyoxylate + 4 Acetyl-CoA → 4 Malate + 4 CoA (equation 6)

4 Succinate + 4 FAD^+^ → 4 Fumarate + 4 FADH_2_ (equation 7)

4 Fumarate + 4 H_2_O → 4 Malate (equation 8)

8 Malate + 8 NAD^+^ → 8 Oxaloacetate + 8 NADH   (equation 9)

4 Oxaloacetate + 4 ATP → 4 Phosphoenolpyruvate + 4 ADP + 4 CO_2_

(equation 10)

8 Acetate + 8 H_2_O + 20 ATP + 8 NAD^+^ + 4 FAD^+^ →

4 Phosphoenolpyruvate + 4 CO_2_ + 20 ADP + 16 P_i_ + 8 NADH + 4 FADH_2_ (equation 11)

***From 2 mol of PEP to 1 mol of E-4-P***

2 Phosphoenolpyruvate + 2 H_2_O → 2 2-phosphoglycerate + 2 P_i_ (equation 12)

2 2-Phosphoglycerate → 2 3-Phosphoglycerate (equation 13)

2 3-Phosphoglycerate + 2 H_2_O → 2 1,3-Bisphosphoglycerate + 2 P_i_ (equation 14)

2 1,3-bisphosphoglycerate + 2 H_2_O → 2 Glyceraldehyde-3-phosphate + 2 P_i_

(equation 15)

2 Glyceraldehyde-3-phosphate → Fructose-1,6-bisphosphate (equation 16)

Fructose-1,6-bisphosphate + H_2_O → Fructose-6-phosphate + P_i_ (equation 17)

Fructose-6-phosphate → Glucose-6-phosphate (equation 18)

Glucose-6-phosphate + NADP^+^→ 6-Phosphoglucolactone + NADPH (equation 19)

6-Phosphoglucolactone + H_2_O → 6-Phosphogluconate (equation 20)

6-Phosphocluconate + NADP^+^ → Ribulose-5-phosphate + CO_2_ + NADPH

(equation 21)

Riboulose-5-phosphate → 0.5 Xylulose-5-phosphate + 0.5 Ribose-5-phosphate

(equation 22)

0.5 Xylulose-5-phosphate + 0.5 Ribose-5-phosphate
→ 0.5 Glyceraldehyde-3-phosphate + 0.5 Sedoheptulose-7-phosphate (equation 23)

0.5 Glyceraldehyde-3-phosphate + 0.5 Sedoheptulose-7-phosphate
→ 0.5 Erythrose-4-phosphate + 0.5 Fructose-6-phosphate (can be recycled)

(equation 24)

2 Phosphoenolpyruvate + 9 H_2_O + 4 NADP^+^

→ Erythrose-4-phosphate + 2 CO_2_ + 4 NADPH + 7 P_i_ (equation 25)

***From 2 mol of PEP & 1 mol of E-4-P to 1 mol of tyrosine***

Phosphoenolpyruvate + Erythrose-4-phosphate + H_2_O

→ 2-Dehydro-3-deoxy-D-arabino-heptonate-7-phosphate + P_i_ (equation 26)

2-Dehydro-3-deoxy-D-arabino-heptonate-7-phosphate → 3-Dehydroquinate + P_i_ (equation 27)

3-Dehydroquinate → 3-Dehydroshikimate + H_2_O (equation 28)

3-Dehydroshikimate + NADPH → Shikimate + NADP^+^ (equation 29)

Shikimate + ATP → Shikimate-3-phosphate + ADP (equation 30)

Shikimate-3-phosphate + Phosphoenolpyruvate →

5-Enolpyruvylshikimate-3-phosphate + P_i_ (equation 31)

5-Enolpyruvylshikimate-3-phosphate → Chorismate + P_i_ (equation 32)

Chorismate → Prephenate (equation 33)

Prephenate + NAD^+^ → 0.5 2,3-(4-Hydroxyphenyl)-pyruvate + CO_2_ + NADH

(equation 34)

0.5 2,3-(4-Hydroxyphenyl)-pyruvate + Glutamate → Tyrosine + 2-oxoglutarate

(equation 35)

2-Oxoglutarate + NADPH → Glutamate + NADP^+^ (equation 36)

2 Phosphoenolpyruvate + Erythrose-4-phosphate + ATP + NAD^+^ + 2 NADPH

→ Tyrosine + CO_2_ + ADP + 4 P_i_ + NADH + 2 NADP^+^ (equation 37)

***From 8 mol of acetate to 1 mol of tyrosine***

8 Acetate + 17 H_2_O + 21 ATP + 9 NAD^+^ + 4 FAD^+^ + 2 NADP^+^

→ Tyrosine + 7 CO_2_ + 21 ADP + 23 P_i_ + 9 NADH + 4 FADH_2_ + 2 NADPH

(equation 38)

***From 1 mol of glucose to 2 mol of PEP***

Glucose + ATP → Glucose-6-phosphate + ADP (equation 39)

Glucose-6-phosphate → Fructose-6-phosphate (equation 40)

Fructose-6-phosphate + ATP → Fructose-1,6-bisphosphate + ADP (equation 41)

Fructose-1,6-bisphosphate → Glyceraldehyde-3-phosphate (equation 42)

Fructose-1,6-bisphosphate → Dihydroxyacetone phosphate (equation 43)

Dihydroxyacetone phosphate → Glyceraldehyde-3-phosphate (equation 44)

2 Glyceraldehyde-3-phosphate + 2 NAD^+^ + 2 P_i_ → 2 1,3-Bisphosphoglycerate + 2 NADH (equation 45)

2 1,3-Bisphosphoglycerate + 2 ADP → 2 3-Phosphoglycerate + 2 ATP

(equation 46)

2 3-Phosphoglycerate → 2 2-Phosphoglycerate (equation 47)

2 2-Phosphoglycerate → 2 Phosphoenolpyruvate + 2 H_2_O (equation 48)

Glucose + 2 NAD^+^ + 2 P_i_ → 2 Phosphoenolpyruvate + 2 NADH + 2 H_2_O (equation 49)

***From 1 mol of glucose to 1 mol of E-4-P***

Glucose + ATP → Glucose-6-phosphate + ADP (equation 39)

Glucose-6-phosphate + NADP^+^→ 6-Phosphoglucolactone + NADPH (equation 19)

6-Phosphoglucolactone + H_2_O → 6-Phosphogluconate (equation 20)

6-Phosphocluconate + NADP^+^ → Ribulose-5-phosphate + CO_2_ + NADPH

(equation 21)

Riboulose-5-phosphate → 0.5 Xylulose-5-phosphate + 0.5 Ribose-5-phosphate

(equation 22)

0.5 Xylulose-5-phosphate + 0.5 Ribose-5-phosphate
→ 0.5 Glyceraldehyde-3-phosphate + 0.5 Sedoheptulose-7-phosphate (equation 23)

0.5 Glyceraldehyde-3-phosphate + 0.5 Sedoheptulose-7-phosphate
→ 0.5 Erythrose-4-phosphate + 0.5 Fructose-6-phosphate (can be recycled)

(equation 24)

Fructose-6-phosphate → Glucose-6-phosphate (equation 18)

Glucose + 2 ATP + 4 NADP^+^ + 2 H_2_O
→ Erythrose-4-phosphate + 2 CO_2_ + 2 ADP + 4 NADPH (equation 50)

***From 2 mol of glucose to 1 mol of tyrosine***

2 Glucose + 3 ATP + 3 NAD^+^ + 2 NADP^+^

→ Tyrosine + 3 CO_2_ + 3 ADP + 2 P_i_ + 3 NADH + 2 NADPH (equation 51)

**Supplementary reference**

1. Olson MM, Templeton LJ, Suh W, Youderian P, Sariaslani FS, Gatenby AA, Van Dyk TK. Production of tyrosine from sucrose or glucose achieved by rapid genetic changes to phenylalanine-producing *Escherichia coli* strains. Appl Microbial Biotechnol. 2007;74:1031-1040.
2. Muñoz AJ, Hernández-Chávez G, de Anda R, Martínez A, Bolívar F, Gosset G. Metabolic engineering of *Escherichia coli* for improving L-3,4-dihydroxyphenylalanine(L-DOPA) synthesis from glucose. J Ind Microbiol Biotechnol. 2011;38:1845-1852.
3. Santos CN, Stephanopoulos G. Melanin-based high throughput for L-tyrosine production in *Escherichia coli*. Appl Environ Microbiol. 2008;74(4):1190-1197.
4. Lütke-Eversloh T, Stephanopoulos G. Combinatorial pathway analysis for improved L-tyrosine production in *Escherichia coli*: Identification of enzymatic bottlenecks by systematic gene overexpression. 2008;10(2):69-71.
5. Juminaga D, Baidoo EEK, Redding-Johanson AM, Batth TS, Burd H, Mukhopadhyay A, et al. Modular engineering of L-tyrosine production in *Escherichia coli*. Appl Environ Microbiol. 2012;78:89–98.
6. Chávez-Béjar MI, Lara AR, López H, Hernández-Chávez G, Martinez A, Ramírez OT, Bolívar F, Gosset G. Metabolic engineering of *Escherichia coli* for L-tyrosine production by expression of genes coding for the chorismate mutase domain of the native chorismate mutase-prephenate dehydratase and a cyclohexadienyl dehydrogenase from *Zymomonas mobilis*. Appl Environ Microbiol. 2008;74(10):3284-3290.
7. Kim SC, Min BE, Hwang HG, Seo SW, Jung GY. Pathway optimization by re-design of untranslated regions for L-tyrosine production in *Escherichia coli*. Sci Rep. 2015;5:13853.
8. Takai A, Nishi R, Joe YJ, Ito H. L-Tyrosine-producing bacterium and a method for producing L-tyrosine. U.S. Patent No. EP 1 616 940 B1. 2013;1-52.
9. Lütke-Eversloh T, Stephanopoulos G. L-Tyrosine production by deregulated strains of *Escherichia coli.* Appl Microbiol Biotechnol. 2007;75:103–10.
10. Santos CN, Xiao W, Stephanopoulos G. Rational, combinatorial, and genomic approaches for engineering L-tyrosine production in *Escherichia coli.* Proc Natl Acad Sci. U.S.A. 2012;109(34)13538-13543.
11. Patnaik R, Zolandz RR, Green DA, Kraynie DF. L-Tyrosine production by recombinant *Escherichia coli*: Fermentation optimization and recovery. Biotechnol Bioeng. 2008;99(4):741-752.
12. Seo SW, Yang J-S, Kim I, Yang J, Min BE, Kim S, et al. Predictive design of mRNA translation initiation region to control prokaryotic translation efficiency. Metab Eng. 2013;15:67–74.
